# Synthesis of tricarbonylated propargylamine and conversion to 2,5-disubstituted oxazole-4-carboxylates

**DOI:** 10.3762/bjoc.20.238

**Published:** 2024-11-06

**Authors:** Kento Iwai, Akari Hikasa, Kotaro Yoshioka, Shinki Tani, Kazuto Umezu, Nagatoshi Nishiwaki

**Affiliations:** 1 School of Engineering Science, Kochi University of Technology, Tosayamada, Kami, Kochi 782-8502, Japan; 2 Research Center for Molecular Design, Kochi University of Technology, Tosayamada, Kami, Kochi 782-8502, Japan; 3 Department of Chemistry, Faculty of Science, Nara Women’s University, Kitauoyahigashimachi, Nara 630-8506, Japan; 4 Kumiai Chemical Industry Co. Ltd., Nakanogo, Fuji, Shizuoka 421-3306, Japan and 5K • I Chemical Industry Co. Ltd., Shinoshinden, Iwata, Shizuoka 437-1213, Japan

**Keywords:** acid amide, diethyl mesoxalate, *N*-acylamine, oxazole, propargylamine

## Abstract

The *N*,*O*-acetal derived from diethyl mesoxalate (DEMO) undergoes elimination of acetic acid upon treatment with a base, leading to the formation of *N*-acylimine in situ. Lithium acetylide readily attacks the imino group to afford *N*,1,1-tricarbonylated propargylamines. When the resulting propargylamine reacts with butyllithium, ring closure occurs between the ethynyl and carbamoyl groups, yielding 2,5-disubstituted oxazole-4-carboxylates. This cyclization also occurs when the propargylamine is heated with ammonium acetate, resulting in double activation.

## Introduction

**Scheme 1:**
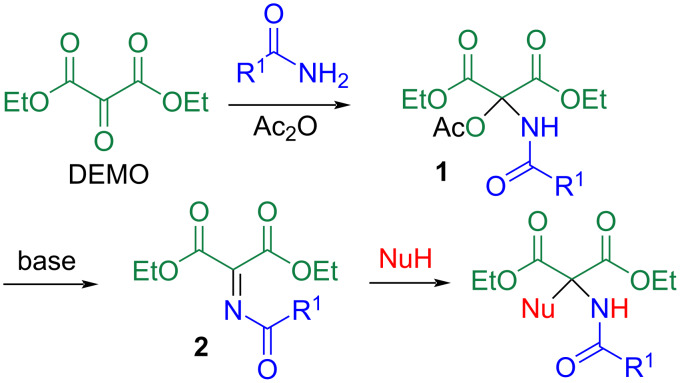
Synthesis of polyfunctionalized methane derivatives through successive nucleophilic additions to the central carbon atom of DEMO.

Propargylamine is an important motif in the synthesis of heterocyclic compounds [1–4] and drug discovery [5–6] due to its multifunctionality, which includes a basic and nucleophilic amino group, an electrophilic and dipolarophilic triple bond, and an acidic propargyl methylene group. Among these, polycarbonylated propargylamines (PCPAs), specifically *N*,1-dicarbonylated or *N*,1,1-tricarbonylated propargylamines, are often used as model compounds to identify biologically active compounds [7–10] or their synthetic precursors [11–15] because of their easily modifiable dipeptide frameworks. Several methods exist for accessing PCPAs, such as the amination of 1-halo-1-alkynes [16–17], tandem reactions of α-imino esters with nucleophiles and electrophiles [18], and the nucleophilic addition of an acetylide to α-carbonylated *N*-acylimines (NAIs) [13–14,19–22]. To apply the latter method to the preparation of *N*,1,1-tricarbonylated propargylamines, the corresponding NAIs are necessary. However, the poor structural diversity of available NAIs limits the use of this method. For example, *N*,1-dicarbonylated NAIs derived from α-keto esters and *N*-acyl groups is limited to acetyl, benzoyl, and alkoxycarbonyl groups [15]. Conversely, NAIs derived from α-oxomalonic acid diester are more versatile. Nagao et al. prepared NAI **2** through the aza-Wittig reaction of diethyl mesoxalate (DEMO, diethyl α-oxomalonate). However, only the *N*-acetyl derivative has been employed (Scheme 1) [13–14].

Recently, we have demonstrated that the central carbonyl group of DEMO is highly electrophilic, facilitating the nucleophilic addition of less reactive reagents such as acid amides [23–26]. When the reaction was conducted in the presence of acetic anhydride, the intermediately formed hemiacetal underwent acetylation, leading to *N*,*O*-acetals **1**. In this method, an acid amide can be used as an amine masked with an acyl group. Subsequent elimination of acetic acid occurred to afford **2** in situ upon treatment with a base, enabling nucleophilic addition with various nucleophiles. This is because the imino carbon atom of **2** is also highly electrophilic, similar to DEMO [23–25]. This method offers an advantage over conventional methods as the *N*-acyl group can be modified by altering the acid amide. In this study, lithium acetylides were employed as nucleophiles to synthesize PCPAs, and their ring closures were also investigated.

## Results and Discussion

NAI **2** can be generated by treating *N*,*O*-acetal **1** with a base, such as triethylamine. However, the addition of an amine was omitted because lithium acetylide functions both as nucleophile and base. When **1a** was reacted with lithium acetylide, which was prepared from ethynylbenzene (**3a**) and butyllithium at 0 °C, the solution turned black, resulting in a complex reaction mixture (Table 1, entry 1). This complication persisted even when the reaction was conducted at −78 °C and then warmed to room temperature without addition of acetic acid. To address this, the reaction was performed at −78 °C, and acetic acid was added at the same temperature, yielding adduct **4a** in 13% yield (Table 1, entry 2). The reaction yield was significantly influenced by the amounts of **3a** and butyllithium used. The yield of **4a** increased when a slight excess of acetylide was used (Table 1, entry 3). Solvation of lithium acetylide was also a critical factor in this reaction (Table 1, entry 4). Therefore, the reaction conditions in Table 1, entry 3 were determined to be optimal.

**Table 1:** Optimization of the reaction conditions for the reaction of **1a** with the lithium acetylide of **3a**.

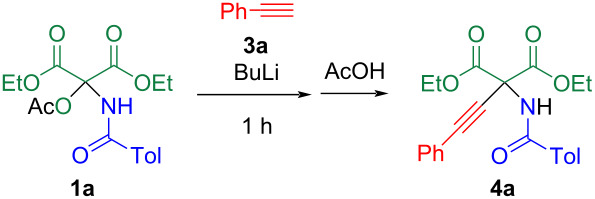					

entry	**3a**, equiv	BuLi , equiv	solvent	temperature, °C	yield, %

1	2	2	THF	0	complex mixture
2	2	2	THF	−78	13
3	2.5	2.15	THF	−78	78
4	2.5	2.15	Et_2_O	−78	no reaction

The optimized conditions were applied to various *N*,*O*-acetals **1** and alkynes **3** to determine the substrate scope of this protocol (Table 2). This reaction was effective with alkyl- and silyl-substituted alkynes **3b**–**d**, yielding the corresponding adducts **4b**–**d,** respectively (Table 2, entries 1–3). A significant advantage of this method is the high modifiability of the *N*-acyl group, achieved by altering the acid amide during the reaction with DEMO [23–25]. Specifically, aliphatic amide **1b** can be used, which reacts with lithium acetylide (**3a**) to yield **4e** (Table 2, entry 4).

**Table 2:** Addition reaction using other *N,O*-acetals **1** and alkynes **3**.

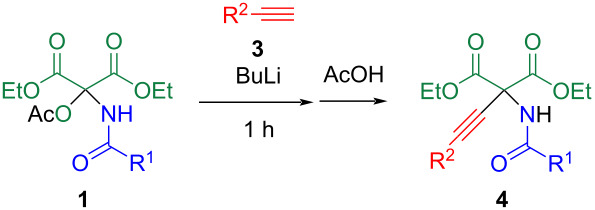						

entry	R^1^	compound	R^2^	compound	product	yield, %

1	*p*-MeC_6_H_4_	**1a**	Bu	**3b**	**4b**	58
2	*p*-MeC_6_H_4_	**1a**	*t*-Bu	**3c**	**4c**	55
3	*p*-MeC_6_H_4_	**1a**	Me_3_Si	**3d**	**4d**	69
4	Et	**1b**	Ph	**3a**	**4e**	72

Subsequently, ring closure utilizing the multifunctionality of **4** was examined (Table 3) [13–14]. To a dry THF solution of adduct **4a**, butyllithium was added, and the reaction mixture was stirred at −78 °C for 5 min. Following the addition of acetic acid, the reaction mixture was concentrated and subjected to silica gel column chromatography, resulting in the isolation of ethyl 5-benzyl-2-(4-methylphenyl)oxazole-4-carboxylate (**5a**) in 13% yield (Table 3, entry 1). Several conditions were considered, but the yield of **5a** did not improve. On the other hand, the addition of two equivalents of water increased the yield, indicating the important role of a stoichiometric amount of water (Table 3, entry 2). Indeed, using THF that had not been dried as purchased resulted in a significant increase of the yield to 82% (Table 3, entry 3). The choice of base was also crucial (Table 3, entries 3–5). While the yield of **5a** was low with potassium *tert*-butoxide, a yield of 66% using lithium *tert*-butoxide was observed, suggesting that lithium ions activate for the ring closure. Under the conditions in Table 3, entry 3, adduct **4** underwent ring formation. This reaction was not influenced by the bulkiness of the substituent on the alkynyl group, yielding the corresponding oxazoles **5b**–**d** (Table 3, entries 6–8). When *N*-propanoyl derivative **4e** was used, cyclization proceeded similarly, yielding the corresponding oxazole **5e** (Table 3, entry 9).

**Table 3:** Conversion of adducts **4** to oxazoles **5**.

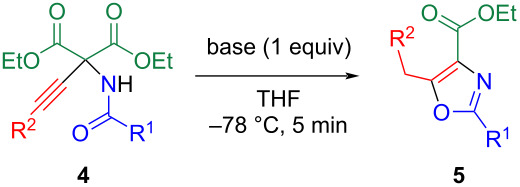					

entry	R^1^	R^2^	base	product	yield, %

1^a^	*p*-MeC_6_H_4_	Ph	BuLi	**5a**	13
2^a,b^	*p*-MeC_6_H_4_	Ph	BuLi	**5a**	24
3	*p*-MeC_6_H_4_	Ph	BuLi	**5a**	82
4	*p*-MeC_6_H_4_	Ph	*t*-BuOK	**5a**	27
5	*p*-MeC_6_H_4_	Ph	*t*-BuOLi	**5a**	66
6	*p*-MeC_6_H_4_	Bu	BuLi	**5b**	45
7	*p*-MeC_6_H_4_	*t*-Bu	BuLi	**5c**	56
8	*p*-MeC_6_H_4_	Me_3_Si	BuLi	**5d**	52
9	Et	Ph	BuLi	**5e**	70

^a^Dry THF was used as solvent; ^b^2 equiv of H_2_O were added.

**Scheme 2:**
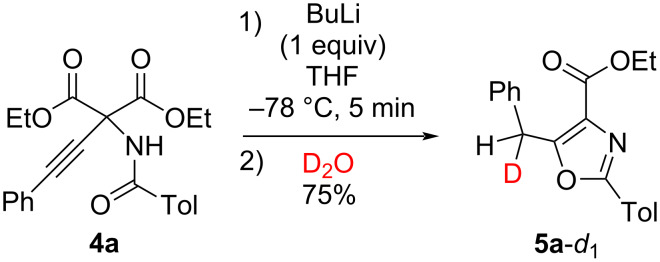
Cyclization of **4a** quenched by D_2_O.

**Scheme 3:**
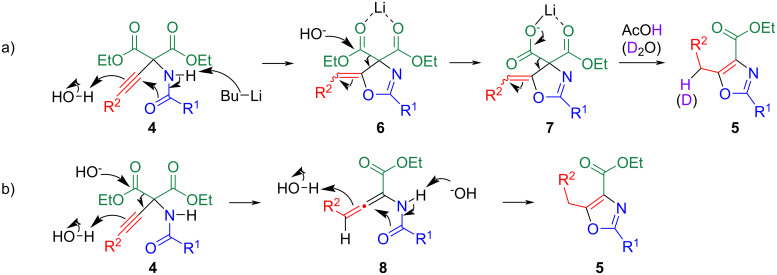
Plausible mechanisms for the ring closure of **4**.

When the reaction was quenched with deuterium oxide instead of acetic acid, monodeuterated oxazole **5a**-*d*_1_ was obtained (Scheme 2). Based on these experimental results, a plausible mechanism was proposed, as shown in Scheme 3a. The 5-*exo*-*dig* ring closure is induced by O-attack of the amide moiety on the ethynyl group to form **6**, during which a stoichiometric proton source (water in the solvent) is necessary. Subsequently, one of the ethoxycarbonyl groups at the 4-position is hydrolyzed to afford lithium carboxylate **7**. In this step, the counter metal ion is considered to affect the activation of the ethoxycarbonyl group of **6**. When the reaction mixture was warmed without the addition of acetic acid, a color change to black was observed, suggesting that decarboxylation accompanied by aromatization of the oxazole ring occurred during this process. Thus, protonation occurs, leading to oxazole **5** when the reaction mixture is warmed in the presence of large amounts of proton sources such as acetic acid or deuterium oxide. Although Nagao et al. proposed another mechanism, as illustrated in Scheme 3b [13–14], we cannot negate this mechanism because the reaction media and bases were different.

**Scheme 4:**
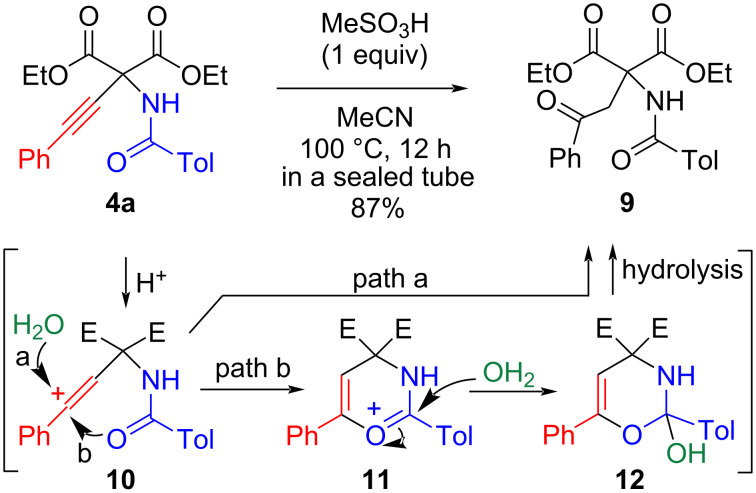
Hydration of the ethynyl group of **4a**.

PCPA **4a** was heated in the presence of methanesulfonic acid to undergo 6-*endo*-*dig* cyclization. However, hydration predominantly occurred, converting the ethynyl group to a phenacyl group, yielding **9** without any detectable cyclization product (Scheme 4). This hydration process is thought to proceed via two paths. The reaction is initiated by the protonation of the ethynyl group to generate the vinyl cation intermediate **10**. Product **9** is directly formed by the attack of a water molecule on this cation, followed by tautomerism (path a). The intramolecular attack of an amide carbonyl on this cationic site in intermediate **10**, leading to the formation of oxonium ion **11**, is also possible (path b). After the addition of water, the formed hemiacetal **12** was hydrolyzed to give the hydrated product **9**.

The less acidic ammonium acetate was effective for the ring closure of **4a** (Table 4). When a solution of **4a** and ammonium acetate was heated for 15 h, a 28% yield of **5a** was obtained (Table 4, entry 1). Ammonium acetate dissociates into ammonia and acetic acid in an equilibrium upon heating, acting both as base and acid. This dual role activates the amide moiety and the ethynyl group, respectively. Using larger amounts of ammonium acetate substantially prolongs the reaction time due to its dissociation properties. Consequently, the yield of **5a** increased to 92% by increasing the amount of ammonium acetate and extending the reaction time (Table 4, entries 2 and 3).

**Table 4:** Ring closure of **4a** using ammonium acetate.

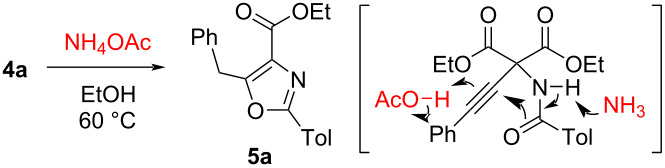			

entry	NH_4_OAc, equiv	time, h	yield, %

1	5	15	28
2	10	15	55
3	10	60	92

## Conclusion

*N*,*O*-Acetals **1**, derived from DEMO and acid amides, reacted with lithium acetylide to afford the corresponding adduct **4** through highly electrophilic NAIs. The *N*-acyl and alkynyl groups could be modified using acid amides and acetylides, respectively. When adduct **4** was treated with a base or ammonium acetate, ring closure proceeded to form a five-membered ring, accompanied by the elimination of the ethoxycarbonyl group.

2,5-Disubstituted oxazole-4-carboxylic acid derivatives are frequently found in biologically active compounds [27–31] and their synthetic intermediates [32–36]. Thus, this method, which enables modification at the 2- and 5-positions of oxazole-4-carboxylates, is a valuable tool for the study of these compounds.

## Experimental

### General

All reagents except for DEMO were purchased from commercial sources (Kanto Chemical Co., Inc. or Fujifilm Wako Pure Chemical Corp.) and used without further purification. Super dehydrated, stabilizer-free THF was used as solvent and purchased from Fujifilm Wako Pure Chemical Corp. DEMO was supplied by Kumiai Chemical Industry Co. Ltd. and purified by distillation. ^1^H and ^13^C{^1^H} NMR spectra were recorded on a JEOL JMN-ECZ400S spectrometer (400 MHz and 100 MHz, respectively) using TMS as internal standard. The assignments of the ^13^C{^1^H} NMR signals were reaffirmed by DEPT experiments. IR spectra were recorded with a JASCO FT/IR-4200 spectrometer equipped with an ATM detector. High-resolution mass spectra (HRMS) were obtained with a Bruker compact mass spectrometer APCI–TOF set at positive mode. The melting point was measured on an SRS-OptiMelt automated melting point system.

### Preparation of *N*,*O*-acetal **1**

In a manner analogous to that reported in reference [23], to a solution of DEMO (1.72 g, 10.0 mmol) in toluene (40 mL) were added 4-methylbenzamide (1.63 g, 12 mmol), 3 Å molecular sieves (3.4 g), and acetic anhydride (2.0 mL, 20 mmol). The resulting solution was heated at 100 °C for 4 h. After cooling to room temperature, the molecular sieves were filtered off, and the filtrate was washed with water (50 mL × 2). The organic layer was dried over magnesium sulfate and concentrated under reduced pressure to afford diethyl α-acetoxy-α-(4-methylbenzoylamino)malonate (**1a**, 2.86 g, 8.1 mmol, 81% yield) as white solid. When amide **1b** was used, the experiment was conducted in the same way.

### Synthesis of tricarbonylated propargylamines **4**

Under argon atmosphere, a solution of ethynylbenzene (**3a**, 110 μL, 1.0 mmol) in THF (1 mL) was cooled to −50 °C. To this solution, a 1.6 M hexane solution of butyllithium (550 μL, 0.86 mmol) was added dropwise to afford lithium acetylide.

To a solution of *N*,*O*-acetal **1a** (140.0 mg, 0.4 mmol) in THF (3 mL), the above-mentioned THF solution of butyllithium was added at −78 °C under argon atmosphere, and the resulting mixture was stirred for a further 1 h. After addition of acetic acid (0.1 mL), the mixture was concentrated under reduced pressure. The residue was purified by column chromatography on silica gel (eluent: hexane/ethyl acetate 70:30, *R*_f_ 0.55) to afford diethyl 2-[(4-methylbenzoyl)amino]-2-(phenylethynyl)propanedioate (**4a**, 122 mg, 0.31 mmol, 78% yield) as yellow oil. ^1^H NMR (400 MHz, CDCl_3_, δ) 7.78 (d, *J* = 8.0 Hz, 2H), 7.65 (br s, 1H), 7.48 (d, *J* = 8.0 Hz, 2H), 7.32–7.25 (m, 5H), 4.37 (q, *J* = 7.2 Hz, 4H), 2.40 (s, 3H), 1.35 (t, *J* = 7.2 Hz, 6H); ^13^C{^1^H} NMR (100 MHz, CDCl_3_, δ) 165.6 (C), 165.3 (C), 142.8 (C), 130.2 (C), 129.4 (CH), 128.9 (CH), 128.2 (CH), 127.5 (CH), 122.0 (C), 84.9 (C), 82.6 (C), 63.8 (CH_2_), 61.0 (C), 21.6 (CH_3_), 14.0 (CH_3_); IR (KBr, ATR) *v*_max_: 1754, 1672, 1477, 1281, 1214, 1071, 751 cm^−1^; HRMS–APCI-TOF (*m*/*z*): [M + H]^+^ calcd for C_23_H_24_NO_5_, 394.1649; found, 394.1672.

When other alkynes and *N,O*-acetals were used, the experiments were conducted in the same way.

### Cyclization of tricarbonylated propargylamine **4** leading to oxazoles **5**

To a solution of propargylamine **4a** (137 mg, 0.35 mmol) in THF (3 mL), 1.6 M butyllithium hexane solution (230 μL, 0.35 mmol) was added at −78 °C under argon atmosphere, and the resulting mixture was stirred for 5 min. After quenching with acetic acid (0.1 mL), the solvent was removed under reduced pressure. The residue was purified by column chromatography on silica gel (eluent: hexane/ethyl acetate 70:30, eluent for TLC: hexane/ethyl acetate 80:20, *R*_f_ 0.61) to afford ethyl 2-(4-methylphenyl)-5-(phenylmethyl)oxazole-4-carboxylate (**5a**, 92.2 mg, 0.29 mmol, 82% yield) as colorless oil. ^1^H NMR (400 MHz, CDCl_3_, δ) 7.88 (d, *J* = 8.4 Hz, 2H), 7.35–7.22 (m, 7H), 4.45 (s, 2H), 4.42 (q, *J* = 7.2 Hz, 2H), 2.39 (s, 3H), 1.40 (t, *J* = 7.2 Hz, 3H); ^13^C{^1^H} NMR (100 MHz, CDCl_3_, δ) 162.0 (C), 160.7 (C), 157.6 (C), 141.8 (C), 136.4 (C), 129.4 (CH), 128.5 (CH), 128.3 (C), 128.1 (CH), 126.8 (CH), 126.2 (CH), 123.4 (C), 60.9 (CH_2_), 31.6 (CH_2_), 20.2 (CH_3_), 13.3 (CH_3_); IR (KBr, ATR) *v*_max_: 1735, 1710, 1178, 1087, 1054, 720 cm^−1^; HRMS–APCI-TOF (*m*/*z*): [M + H]^+^ calcd for C_20_H_20_NO_3_, 322.1438; found, 322.1458.

When other propargylamines were used, the experiments were conducted in the same way. In the deuteration experiment, the reaction was quenched with deuterium oxide (0.2 mL) instead of acetic acid. The decrease of the integral of the signal of the benzyl proton was confirmed by ^1^H NMR analysis.

## Supporting Information

File 1 Spectral data for **4**, **5**, and **9** as well as ^1^H and ^13^C NMR spectra.

## Data Availability

All data that supports the findings of this study is available in the published article and/or the supporting information of this article.
