# Supplementary material for: Synthesis of tricarbonylated propargylamine and conversion to 2,5-disubstituted oxazole-4-carboxylates
Source: Beilstein J Org Chem. 2024 Nov 6;20:2827–33. doi: 10.3762/bjoc.20.238 (PMC11552412; doi:10.3762/bjoc.20.238)

BEILSTEIN JOURNAL OF ORGANIC CHEMISTRY

Supporting Information

for

Synthesis of tricarbonylated propargylamine and conversion to 2,5-disubstituted oxazole-4-carboxylates

Kento Iwai, Akari Hikasa, Kotaro Yoshioka, Shinki Tani, Kazuto Umezu and Nagatoshi Nishiwaki

Beilstein J. Org. Chem. **2024,** *20,* 2827–2833. doi:10.3762/bjoc.20.238

Spectral data for 4, 5, and 9 as well as ^{1}H and ^{13}C NMR spectra

License and Terms: This is a supporting information file under the terms of the Creative Commons Attribution License (https://creativecommons.org/licenses/by/4.0). Please note that the reuse, redistribution and reproduction in particular requires that the author(s) and source are credited and that individual graphics may be subject to special legal provisions.
The license is subject to the *Beilstein Journal of Organic Chemistry* terms and conditions: (https://www.beilstein-journals.org/bjoc/terms)

Table of contents

General information ·································· **S2**

Experimental methods and spectral data ·································· **S2**

Copies of NMR spectra of propargyl alcohol 4 ·································· **S8**

Copies of NMR spectra of oxazole 5 ·································· **S13**

Copies of NMR spectra of hydrated product 9 ·································· **S19**

General

All reagents except for diethyl mesoxalate (DEMO) were purchased from commercial sources and used without further purification. DEMO was supplied by Kumiai Chemical Industry Co. Ltd. and purified by distillation. ^{1}H and ^{13}C{^{1}H} NMR spectra were recorded on a JEOL JMN-ECZ400S spectrometer (400 MHz and 100 MHz, respectively) using TMS as an internal standard. The assignments of the ^{13}C{^{1}H} NMR were reaffirmed by DEPT experiments. IR spectra were recorded with a JASCO FT/IR-4200 spectrometer equipped with an ATM detector. High-resolution mass spectra (HRMS) data were obtained from a Bruker compact mass spectrometer APCI–TOF set at positive mode. The melting points were measured on an SRS-Optimelt automated melting point system.

Preparation of *N,O*-acetal 1

In a manner analogous to [*J. Org. Chem.* **2023**, *88*, 2207–2213]. To a solution of DEMO (1.72 g, 10.0 mmol) in toluene (40 mL), were added 4-methylbenzamide (1.63 g, 12 mmol), molecular sieves 3 Å (3.4 g) and acetic anhydride (2.0 mL, 20 mmol), and the resultant solution was heated at 100 °C for 4 h. After cooling to room temperature, molecular sieves were filtered off, and the filtrate was washed with water (50 mL × 2). The organic layer was dried over magnesium sulfate and concentrated under reduced pressure to afford diethyl α-acetoxy-α-(4-methylbenzoylamino)malonate (**1a**, 2.86 g, 8.1 mmol, yield 81%) as a white solid. When other amides were used, the experiments were conducted in the same way.

Synthesis of tricarbonylated propargylamines 4

Under argon atmosphere, a solution of ethynylbenzene (**3a**, 110 μL, 1.0 mmol) in THF (1 mL) was cooled to −50 °C. To this solution, 1.6 M hexane solution of butyllithium (550 μL, 0.86 mmol) was added dropwise to afford lithium acetylide.

To a solution of *N,O*-acetal **1a** (140.0 mg, 0.4 mmol) in THF (3 mL), the above-mentioned THF solution of butyllithium was added at −78 °C under argon

atmosphere, and the resultant mixture was stirred for further 1 h. After addition of acetic acid (0.1 mL), the mixture was concentrated under reduced pressure. The residue was treated with column chromatography on silica gel (eluent: hexane/ethyl acetate = 70:30, R_f = 0.55) to afford diethyl 2-[(4-methylbennzoyl)amino]-2-(phenylethynyl)propanedioate (**4a**) (122 mg, 0.31 mmol, yield 78%) as an yellow oil. When other alkynes and *N,O*-acetals were used, the experiments were conducted in the same way.

^{1}H NMR (400 MHz, $CDCl_3$) δ 7.78 (d, J = 8.0 Hz, 2H), 7.65 (br s, 1H), 7.48 (d, J = 8.0 Hz, 2H), 7.32–7.25 (m, 5H), 4.37 (q, J = 7.2 Hz, 4H), 2.40 (s, 3H), 1.35 (t, J = 7.2 Hz, 6H); ^{13}C{^{1}H} NMR (100 MHz, $CDCl_3$) δ 165.6 (C), 165.3 (C), 142.8 (C), 130.2 (C), 129.4 (CH), 128.9 (CH), 128.2 (CH), 127.5 (CH), 122.0 (C), 84.9 (C), 82.6 (C), 63.8 (CH_2), 61.0 (C), 21.6 (CH_3), 14.0 (CH_3); IR (ATR, KBr) v = 1754, 1672, 1477, 1281, 1214, 1071, 751 cm^{-1}; HRMS (APCI–TOF) *m/z* calcd. for $C_{23}H_{24}NO_5$ [M+H]$^+$ 394.1649, found 394.1672.

Diethyl 2-(hexyn-1-yl)-2-[(4-methylbenzoyl)amino]propanedioate (**4b**)

Pale yellow oil (87 mg, 0.23 mmol, yield 58%, eluent of TLC: hexane/ethyl acetate = 80:20, R_f = 0.69). ^{1}H NMR (400 MHz, $CDCl_3$) δ 7.75 (d, J = 8.0 Hz, 2H), 7.56 (br s, 1H), 7.24 (d, J = 8.0 Hz, 2H), 4.34 (q, J = 7.6 Hz, 4H), 2.40 (s, 3H), 2.25 (t, J = 7.2 Hz, 2H), 1.50 (tt, J = 6.8, 7.2 Hz, 2H), 1.40 (tq, J = 6.8, 7.6 Hz, 2H), 1.30 (t, J = 6.8 Hz, 6H), 0.88 (t, J = 7.6 Hz, 3H); ^{13}C{^{1}H} NMR (100 MHz, $CDCl_3$) δ 165.7 (C), 165.5 (C), 142.6 (C), 130.3 (C), 129.3 (CH), 127.4 (CH), 86.4 (C), 73.7 (C), 63.6 (CH_2), 60.6 (C), 30.3 (CH_2), 21.9 (CH_2), 21.6 (CH_3), 18.7 (CH_2), 14.0 (CH_3), 13.6 (CH_3); IR (ATR, KBr) v = 2246, 1749, 1478, 1280 cm^{-1}; HRMS (APCI–TOF) *m/z* calcd. for $C_{21}H_{28}NO_5$ [M+H]$^+$ 374.1962, found 374.1968.

Diethyl 2-(3,3-dimethylbut-1-yn-1-yl)-2-[(4-methylbenzoyl)amino]propanedioate (**4c**)

Colorless oil (82 mg, 0.22 mmol, yield 55%, eluent of TLC: hexane/ethyl acetate = 80:20, R_f = 0.49). ^{1}H NMR (400 MHz, $CDCl_3$) δ 7.74 (d, J = 8.0 Hz, 2H), 7.50 (s, 1H), 7.24 (d, J = 8.0 Hz, 2H), 4.33 (qd, J = 7.2, 11.2 Hz, 2H), 4.32 (qd, J = 7.2, 11.2 Hz, 2H), 2.40 (s, 3H), 1.30 (dd, J = 7.2, 7.2 Hz, 6H), 1.22 (s, 9H); ^{13}C{^{1}H} NMR (100 MHz, $CDCl_3$) δ 165.8 (C), 165.4 (C), 142.5 (C), 130.5 (C), 129.3 (CH), 127.4 (CH),

94.2 (C), 72.3 (C), 63.4 (CH_2), 60.5 (C), 30.6 (CH_3), 27.6 (C), 21.6 (CH_3), 13.9 (CH_3); IR (ATR, KBr) ν = 1748, 1676, 1478, 1280, 1205 cm^{-1}; HRMS (APCI–TOF) *m/z* calcd. for $C_{21}H_{28}NO_5$ $[M+H]^+$ 374.1962, found 374.1996.

Diethyl 2-[2-(trimethylsilyl)ethynyl]-2-[(4-methylbenzoyl)amino]propanedioate (**4d**)

Colorless oil (108 mg, 0.28 mmol, yield 69%, eluent of TLC: hexane/ethyl acetate = 80:20, R_f = 0.63). ^{1}H NMR (400 MHz, $CDCl_3$) δ 7.75 (d, *J* = 8.0 Hz, 2H), 7.52 (s, 1H), 7.25 (d, *J* = 8.0 Hz, 2H), 4.35 (qd, *J* = 7.2, 10.8 Hz, 2H), 4.34 (qd, *J* = 7.2, 10.8 Hz, 2H), 2.40 (s, 3H), 1.31 (dd, *J* = 7.2, 7.2 Hz, 6H), 0.17 (s, 9H); $^{13}C\{^1H\}$ NMR (100 MHz, $CDCl_3$) δ 165.4 (C), 165.1 (C), 142.7 (C), 130.3 (C), 129.3 (CH), 127.4 (CH), 97.6 (C), 90.7 (C), 63.7 (CH_2), 61.0 (C), 21.6 (CH_3), 13.9 (CH_3), -0.26 (CH_3); IR (ATR, KBr) ν = 1748, 1480, 1281, 772 cm^{-1}; HRMS (APCI–TOF) *m/z* calcd. for $C_{20}H_{28}NO_5Si$ $[M+H]^+$ 390.1731, found 390.1742.

Diethyl 2-(phenylethynyl)-2-(propanoylamino)propanedioate (**4e**)

Pale yellow oil (96 mg, 0.29 mmol, yield 72%, eluent of TLC: hexane/ethyl acetate = 70:30, R_f = 0.33). ^{1}H NMR (400 MHz, $CDCl_3$) δ 7.47 (d, *J* = 8.0 Hz, 2H), 7.34–7.26 (m, 3H), 6.97 (br s, 1H), 4.35 (qd, *J* = 7.6, 10.8 Hz, 2H), 4.35 (qd, *J* = 7.6, 10.8 Hz, 2H), 2.33 (q, *J* = 7.2 Hz, 2H), 1.33 (dd, *J* = 7.6, 7.6 Hz, 6H), 1.19 (t, *J* = 7.2 Hz, 3H); $^{13}C\{^1H\}$ NMR (100 MHz, $CDCl_3$) δ 172.4 (C), 165.2 (C), 132.2 (CH), 128.9 (CH), 128.2 (CH), 122.0 (C), 84.7 (C), 82.7 (C), 63.8 (CH_2), 60.7 (C), 29.1 (CH_2), 14.0 (CH_3), 9.4 (CH_3); IR (ATR, KBr) ν = 1752, 1676, 1222, 772 cm^{-1}; HRMS (APCI–TOF) *m/z* calcd. for $C_{18}H_{21}NO_5$ $[M+H]^+$ 332.1492, found 332.1481.

Cyclization of tricarbonylated propargylamine 4 leading to oxazoles 5

To a solution of propargylamine **4a** (137 mg, 0.35 mmol) in THF (3 mL), 1.6 M butyllithium hexane solution (230 μL, 0.35 mmol) was added at −78 °C under argon atmosphere, and the resultant mixture was stirred for 5 min. After quenching with acetic acid (0.1 mL), the solvent was removed under reduced pressure. The residue was treated with column chromatography on silica gel (eluent: hexane/ethyl acetate = 70:30, eluent of TLC: hexane/ethylacetate = 80:20, R_f = 0.61) to afford ethyl 2-(4-methylphenyl)-5-(phenylmethyl)oxazole-4-carboxylate (**5a**) (92.2 mg, 0.29

mmol, yield 82%) as a colorless oil. When other propargylamines were used, experimental was conducted in a same way.

^{1}H NMR (400 MHz, CD_3OD) δ 7.88 (d, *J* = 8.4 Hz, 2H), 7.35–7.22 (m, 7H), 4.45 (s, 2H), 4.42 (q, *J* = 7.2 Hz, 2H), 2.39 (s, 3H), 1.40 (t, *J* = 7.2 Hz, 3H); $^{13}C\{^{1}H\}$ NMR (100 MHz, CD_3OD) δ 162.0 (C), 160.7 (C), 157.6 (C), 141.8 (C), 136.4 (C), 129.4 (CH), 128.5 (CH), 128.3 (C), 128.1 (CH), 126.8 (CH), 126.2 (CH), 123.4 (C), 60.9 (CH_2), 31.6 (CH_2), 20.2 (CH_3), 13.3 (CH_3); IR (ATR, KBr) *ν* = 1735, 1710, 1178, 1087, 1054, 720 cm^{-1}; HRMS (APCI–TOF) *m/z* calcd. for $C_{20}H_{20}NO_3$ $[M+H]^+$ 322.1438, found 322.1458.

Ethyl 2-(4-methylphenyl)-5-pentyloxazole-4-carboxylate (**5b**)

Even though purification was attempted several times, impurities could not be removed. Hence, larger numbers of signals were observed at the higher field in the NMR spectra. Yellow oil (48 mg, 0.16 mmol, yield 45%, eluent of TLC: hexane/ethyl acetate = 80:20, R_f = 0.61). ^{1}H NMR (400 MHz, $CDCl_3$) δ 7.96 (d, *J* = 8.4 Hz, 2H), 7.25 (d, *J* = 8.4 Hz, 2H), 4.42 (q, *J* = 7.2 Hz, 2H), 3.09 (t, *J* = 7.2 Hz, 2H), 2.40 (s, 3H), 1.75 (tt, *J* = 7.6, 7.6 Hz, 2H), 1.42 (t, *J* = 7.2 Hz, 3H), 1.4–1.2 (m, 4H), 0.91 (t, *J* = 6.4 Hz, 3H); $^{13}C\{^{1}H\}$ NMR (100 MHz, $CDCl_3$) δ 162.6 (C), 159.98 (C), 159.94 (C), 142.1 (C), 141.1 (C), 129.5 (CH), 128.4 (C), 126.7 (CH), 39.1 (CH_2), 31.4 (CH_2), 27.7 (CH_2), 26.2 (CH_2), 22.4 (CH_2), 21.6 (CH_3), 14.5 (CH_3), 14.0 (CH_3); HRMS (APCI–TOF) *m/z* calcd. for $C_{18}H_{24}NO_3$ $[M+H]^+$ 302.1751, found 302.1778.

Ethyl 5-[(2,2-dimethyl)prop-1-yl]-2-(4-methylphenyl)oxazole-4-carboxylate (**5c**)

White solid (59 mg, 0.20 mmol, yield 56%, eluent of TLC: hexane/ethyl acetate = 70:30, R_f = 0.71), mp 64–67 °C (dec.). ^{1}H NMR (400 MHz, $CDCl_3$) δ 7.96 (d, *J* = 7.6 Hz, 2H), 7.60 (d, *J* = 7.6 Hz, 2H), 4.41 (q, *J* = 7.2 Hz, 2H), 3.03 (s, 2H), 2.40 (s, 3H), 1.42 (t, *J* = 7.2 Hz, 3H), 1.05 (s, 9 H); $^{13}C\{^{1}H\}$ NMR (100 MHz, $CDCl_3$) δ 162.6 (C), 160.2 (C), 158.1 (C), 141.2 (C), 130.0 (C), 129.5 (CH), 126.7 (CH), 124.1 (C), 61.0 (CH_2), 39.3 (CH_2), 32.9 (C), 29.7 (CH_3), 21.6 (CH_3), 14.5 (CH_3); IR (ATR, KBr) *ν* = 2961, 1736, 1715 cm^{-1}; HRMS (APCI–TOF) *m/z* calcd. for $C_{18}H_{24}NO_3$ $[M+H]^+$ 302.1751, found 302.1736.

Ethyl 5-(trimethylsilyl)methyl-2-(4-methylphenyl)oxazole-4-carboxylate (**5d**)

Pale yellow oil (58 mg, 0.18 mmol, yield 52%, eluent of TLC: hexane/ethyl acetate = 80:20, R_f = 0.53). ^{1}H NMR (400 MHz, $CDCl_3$) δ 7.96 (d, J = 8.0 Hz, 2H), 7.24 (d, J = 8.0 Hz, 2H), 4.39 (q, J = 6.4 Hz, 2H), 2.65 (s, 2H), 2.39 (s, 3H), 1.40 (t, J = 6.4 Hz, 3H), 0.11 (s, 9 H); $^{13}C\{^1H\}$ NMR (100 MHz, $CDCl_3$) δ 163.0 (C), 160.0 (C), 159.0 (C), 140.9 (C), 129.5 (CH), 126.4 (CH), 126.3 (C), 124.2 (C), 60.8 (CH_2), 21.6 (CH_3), 18.0 (CH_2), 14.5 (CH_3), -1.35 (CH_3); IR (ATR, KBr) ν = 1707, 1598, 851 cm^{-1}; HRMS (APCI–TOF) *m/z* calcd. for $C_{17}H_{24}NO_3Si$ $[M+H]^+$ 318.1520, found 318.1560.

Ethyl 2-ethyl-5-(phenylmethyl)oxazole-4-carboxylate (**5e**)

Colorless oil (64 mg, 0.25 mmol, yield 70%, eluent of TLC: hexane/ethyl acetate = 70:30, R_f = 0.61). ^{1}H NMR (400 MHz, $CDCl_3$) δ 7.33–7.23 (m, 5H), 4.40 (q, J = 7.2 Hz, 2 H), 4.35 (s, 2H), 2.75 (q, J = 7.6 Hz, 2H), 1.40 (t, J = 7.2 Hz, 3H), 1.30 (t, J = 7.6 Hz, 3H); $^{13}C\{^1H\}$ NMR (100 MHz, $CDCl_3$) δ 164.5 (C), 162.5 (C), 157.3 (C), 136.5 (C), 128.8 (CH), 128.8 (CH), 127.5 (C), 127.0 (CH), 61.1 (CH_2), 32.1 (CH_2), 21.7 (CH_2), 14.5 (CH_3), 11.2 (CH_3); IR (ATR, KBr) ν = 1711, 1198, 1179, 1085, 1057 cm^{-1}; HRMS (APCI–TOF) *m/z* calcd. for $C_{15}H_{18}NO_3$ $[M+H]^+$ 260.1281, found 260.1255.

Hydration of propargylamine 4a leading to 9

To a solution of propargylamine **4a** (209 mg, 0.53 mmol) in acetonitrile (4.5 mL), methanesulfonic acid (53 μL, 0.53 mmol) was added, and the mixture was heated in a sealed tube at 100 °C for 15 h. After concentration, saturated aqueous solution of sodium hydrogen carbonate (20 mL) was added to the residue. After separation, the organic layer was dried over magnesium sulfate and evaporated to afford diethyl 2-benzoylmethyl-2-[(4-methylbenzoyl)amino]propanedioate (**9**) (189 mg, 0.46 mmol, yield 87%) as a colorless oil (eluent of TLC: hexane/ethyl acetate = 80:20, R_f = 0.67).

^{1}H NMR (400 MHz, $CDCl_3$) δ 7.96 (d, J = 8.4 Hz, 2H), 7.78 (br s, 1H), 7.67 (d, J = 8.0 Hz, 2H), 7.56 (t, J = 7.2 Hz, 1H), 7.44 (dd, J = 7.2, 8.4 Hz, 2H), 7.21 (d, J =8.0 Hz, 2H), 4.39 (s, 2H), 4.30 (qd, J = 7.2, 10.8 Hz, 2H), 4.29 (qd, J = 7.2, 10.8 Hz, 2H), 2.37 (s, 3H), 1.24 (dd, J = 7.2, 7.2 Hz, 6H); $^{13}C\{^1H\}$ NMR (100 MHz, $CDCl_3$) δ 197.0

(C), 167.5 (C), 166.5 (C), 142.6 (C), 136.2 (C), 133.8 (CH), 130.5 (C), 129.3 (CH), 128.8 (CH), 128.3 (CH), 127.3 (CH), 64.3 (C), 63.0 (CH_2), 42.5 (CH_2), 21.6 (CH_3), 14.0 (CH_3); IR (ATR, KBr) v = 1744, 1685, 1663, 1521 cm^{-1}; HRMS (APCI–TOF) *m/z* calcd. for $C_{23}H_{26}NO_6$ $[M+H]^+$ 412.1755, found 412.1736.

Copies of NMR spectra

Diethyl 2-[(4-methylbennzoyl)amino]-2-phenylethynylpropanedioate (**4a**)

^{1}H NMR ($CDCl_3$)

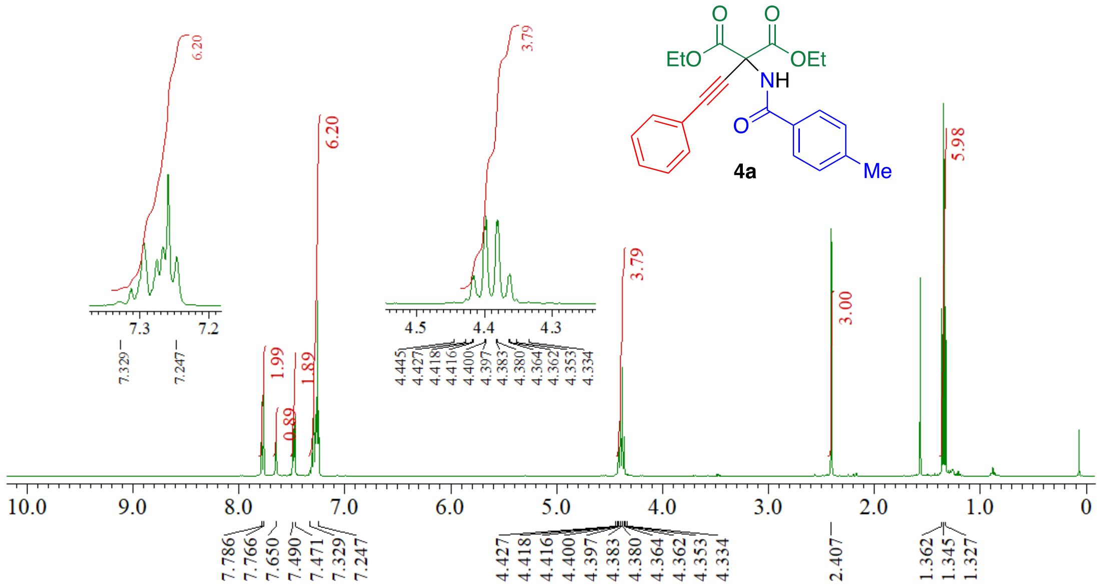

$^{13}C\{^{1}H\}$ NMR ($CDCl_3$)

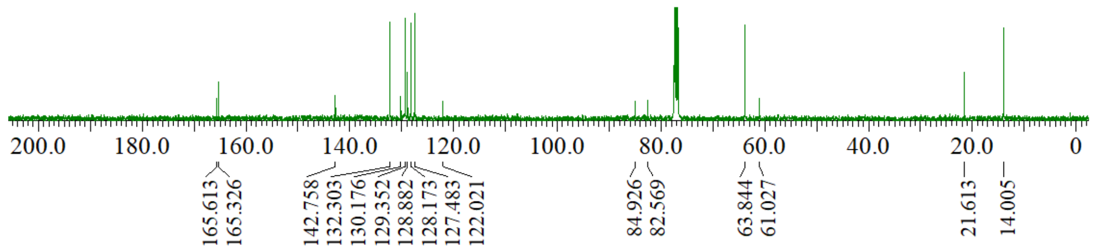

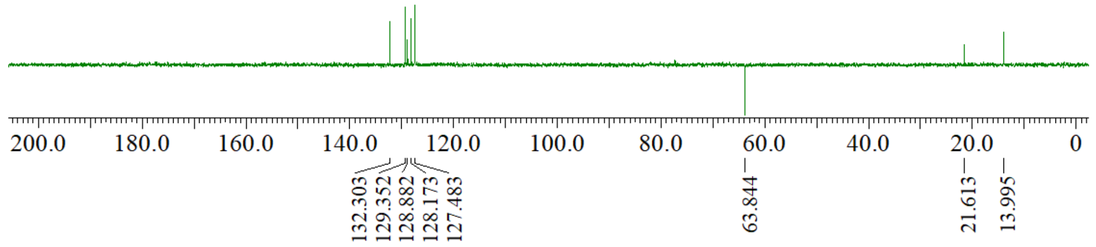

Diethyl 2-(hexyn-1-yl)-2-[(4-methylbenzoyl)amino]propanedioate (**4b**)

^{1}H NMR ($CDCl_3$)

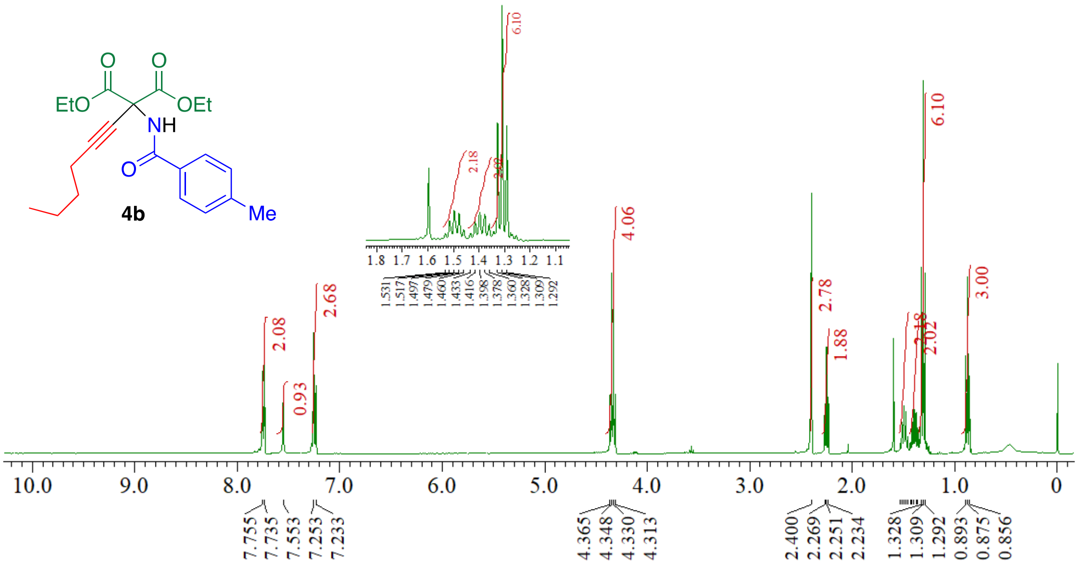

^{13}C{^{1}H} NMR ($CDCl_3$)

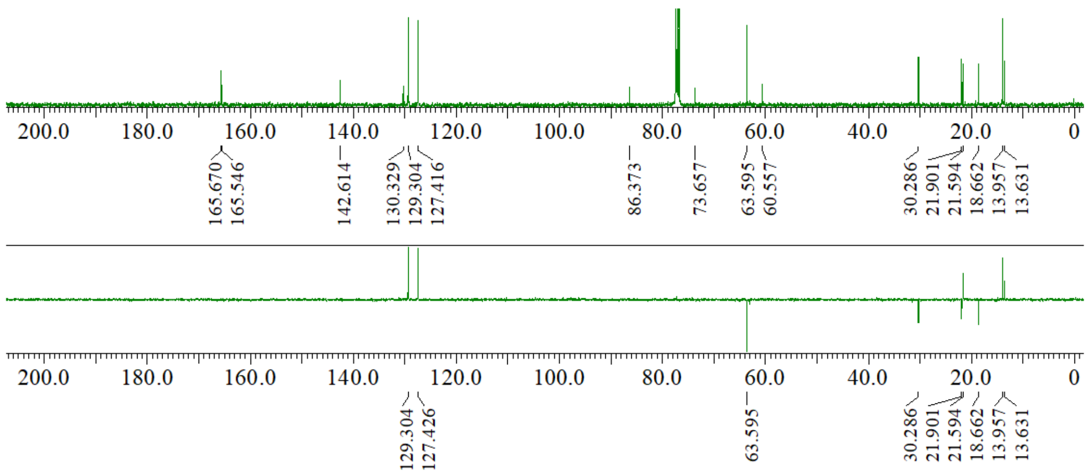

Diethyl 2-(3,3-dimethylbut-1-yn-1-yl)-2-[(4-methylbenzoyl)amino]propanedioate (**4c**)

^{1}H NMR (CDCl$_3$)

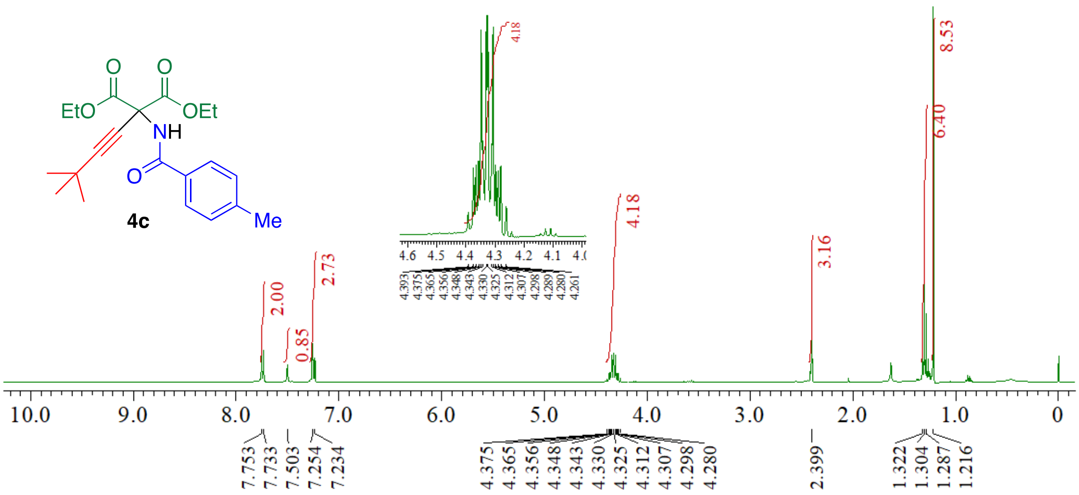

^{13}C{^{1}H} NMR (CDCl$_3$)

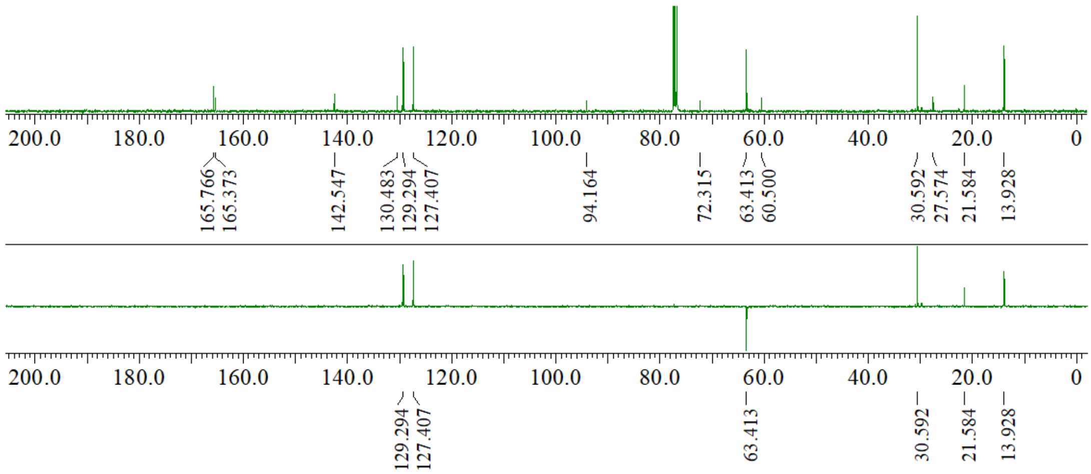

Diethyl 2-[2-(trimethylsilyl)ethynyl]-2-[(4-methylbenzoyl)amino]propanedioate (**4d**)

^{1}H NMR ($CDCl_3$)

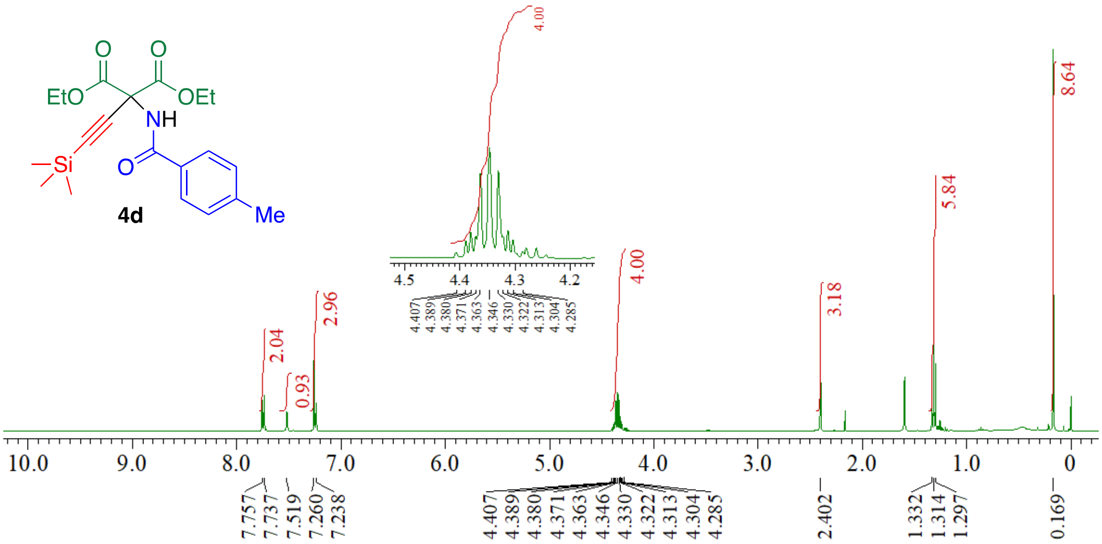

^{13}C{^{1}H} NMR ($CDCl_3$)

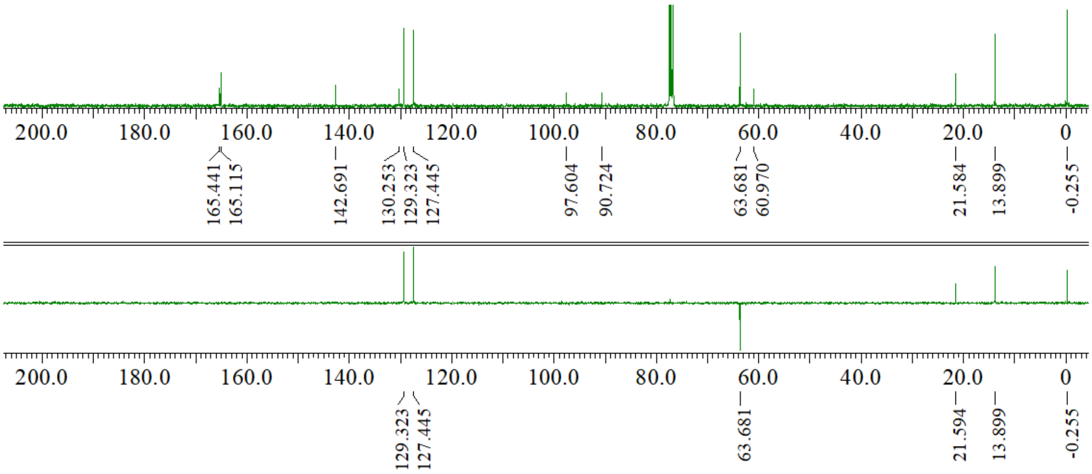

Diethyl 2-(phenylethynyl)-2-(propanoylamino)propanedioate (**4e**)

^{1}H NMR ($CDCl_3$)

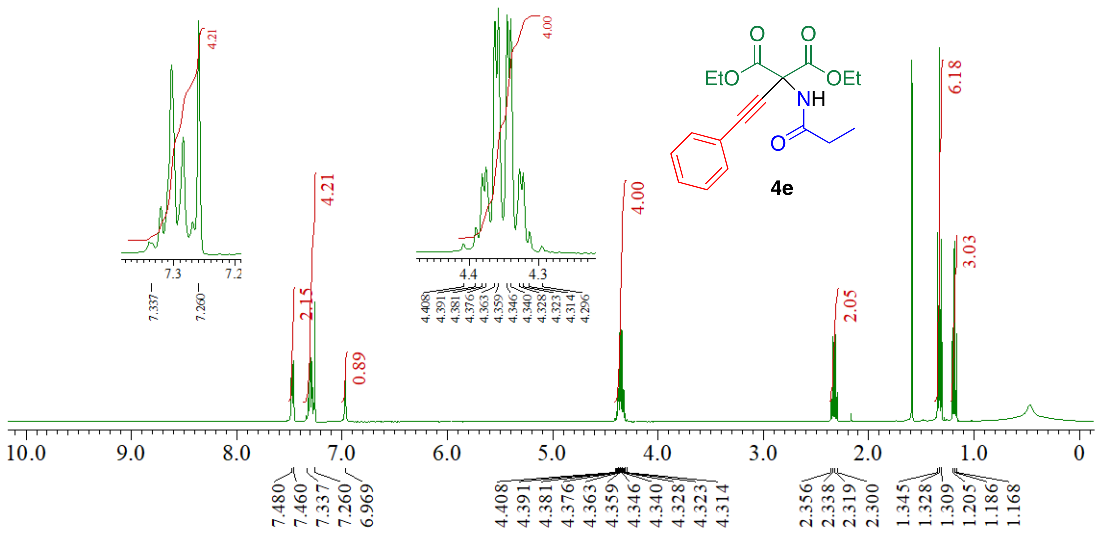

$^{13}C\{^{1}H\}$ NMR ($CDCl_3$)

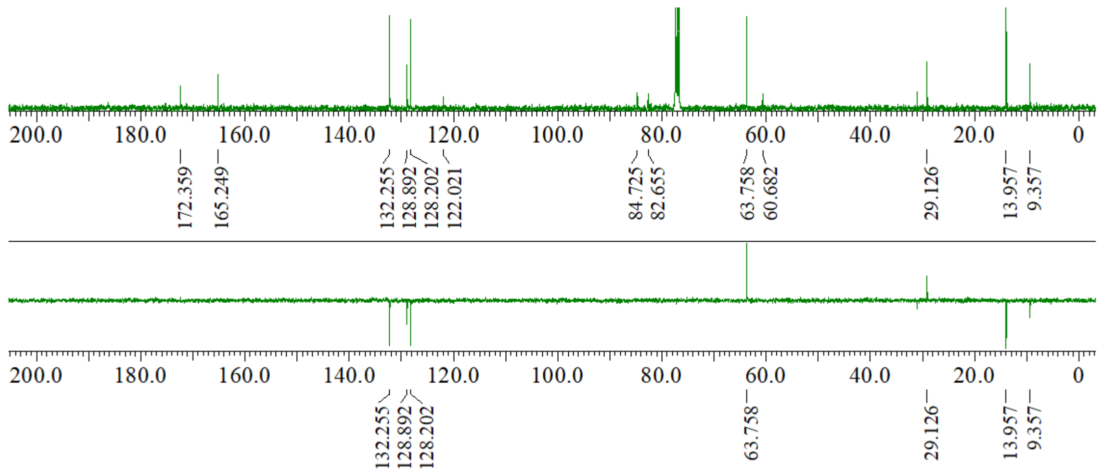

Ethyl 2-(4-methylphenyl)-5-(phenylmethyl)oxazole-4-carboxylate (**5a**)

^{1}H NMR (CD3OD)

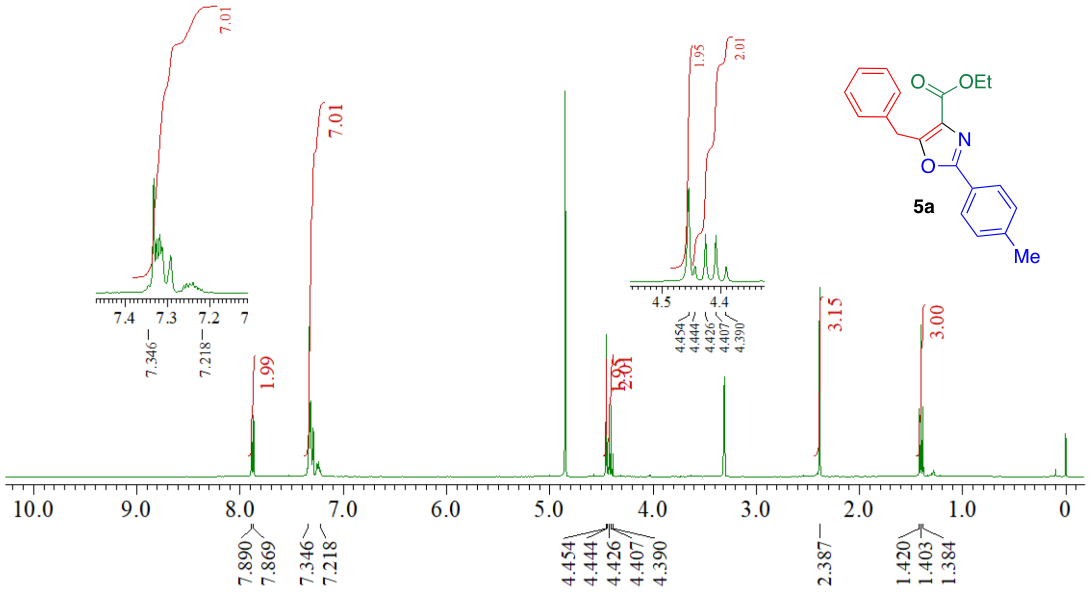

^{13}C{^{1}H} NMR (CD3OD)

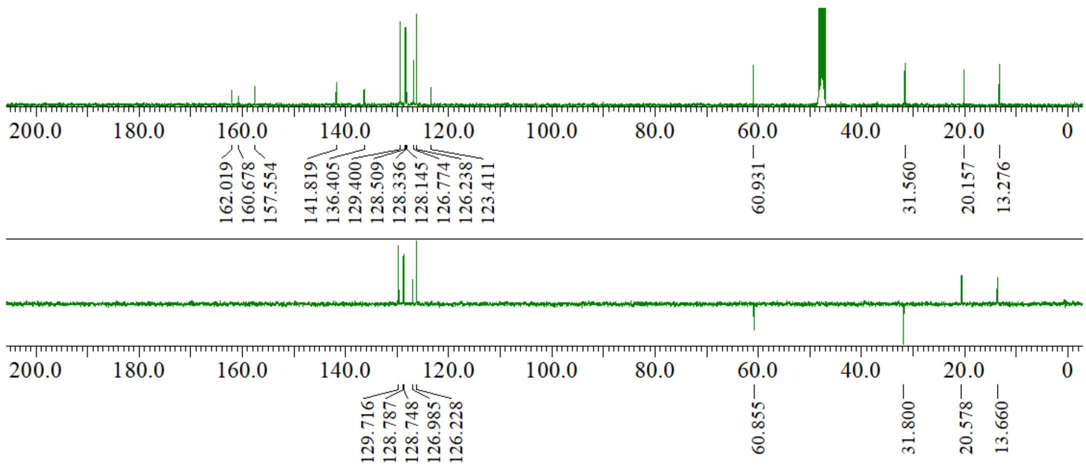

Experiment of deuteration affording **5a**-d_1

Cyclization of **4a** was conducted in the same way except for the quencher; D_2O (0.2 mL) was added to the reaction mixture instead of acetic acid. To confirm whether deuterium was introduced or not, ^1H NMR of the crude mixture was measured to avoid exchange with hydrogen during the purification process.

While benzyl proton was observed as a singlet with 2H integral value in the case of **5a**, the singlet signal becomes smaller with 1H integral value. Hence, we concluded that one deuterium was introduced at the benzyl position.

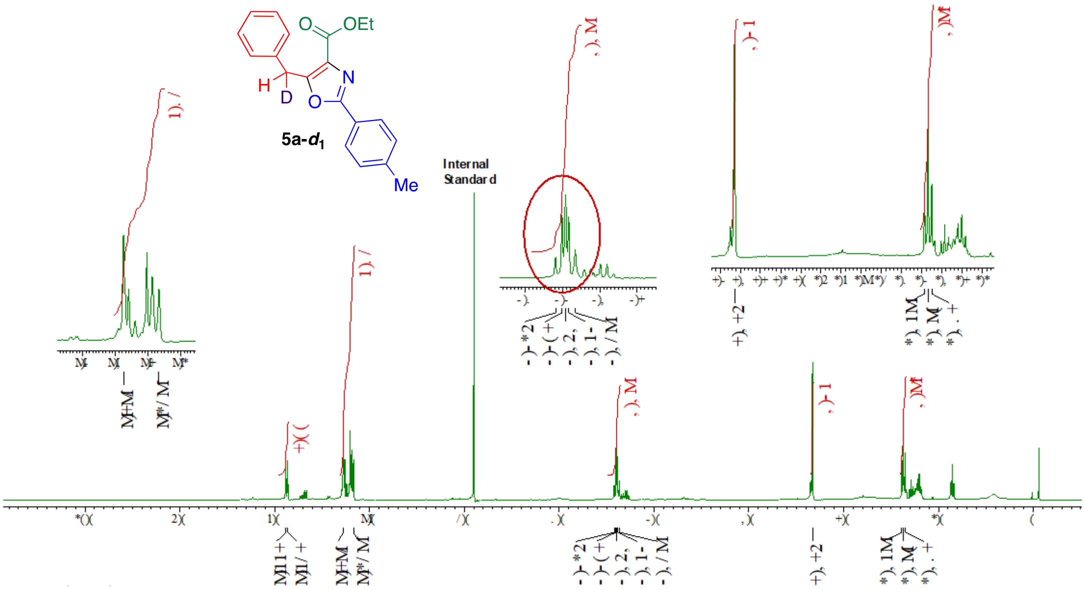

Ethyl 2-(4-methylphenyl)-5-pentyloxazole-4-carboxylate (**5b**)

^{1}H NMR ($CDCl_3$)

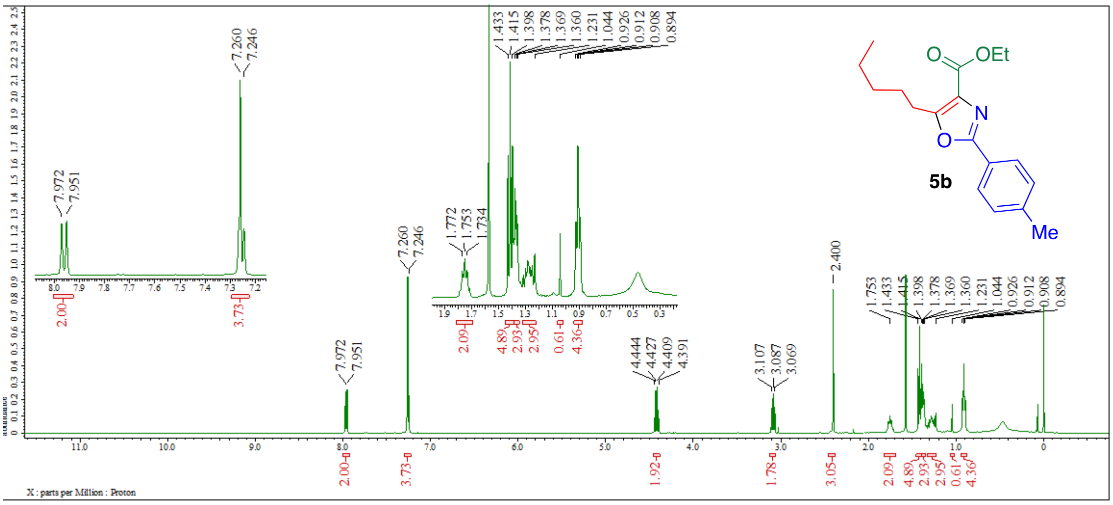

$^{13}C\{^{1}H\}$ NMR ($CDCl_3$)

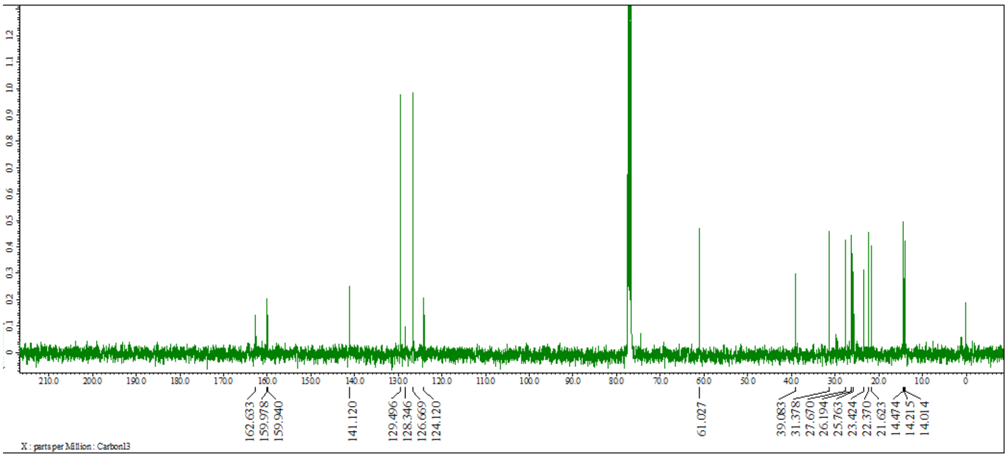

DEPT ($CDCl_3$)

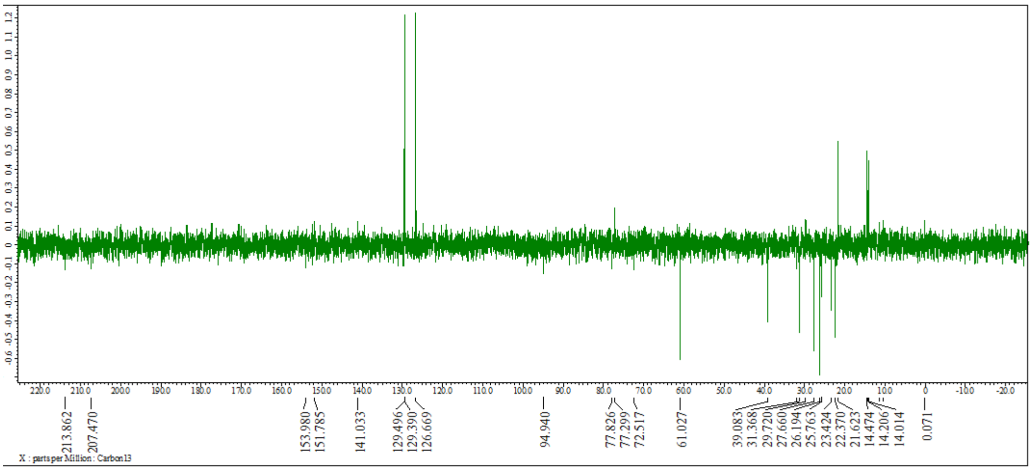

Ethyl 5-[(2,2-dimethyl)prop-1-yl]-2-(4-methylphenyl)oxazole-4-carboxylate (**5c**)

^{1}H NMR ($CDCl_3$)

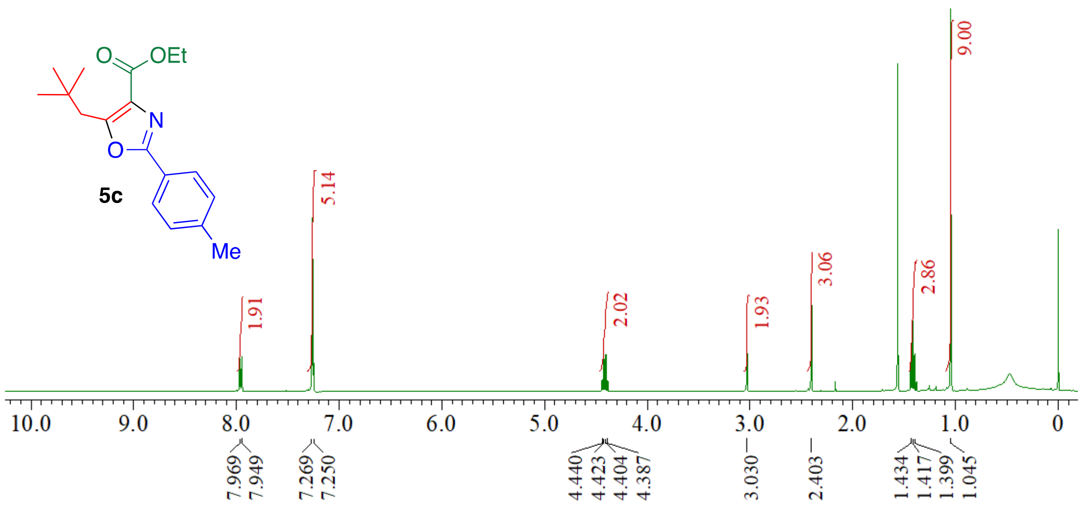

^{13}C{^{1}H} NMR ($CDCl_3$)

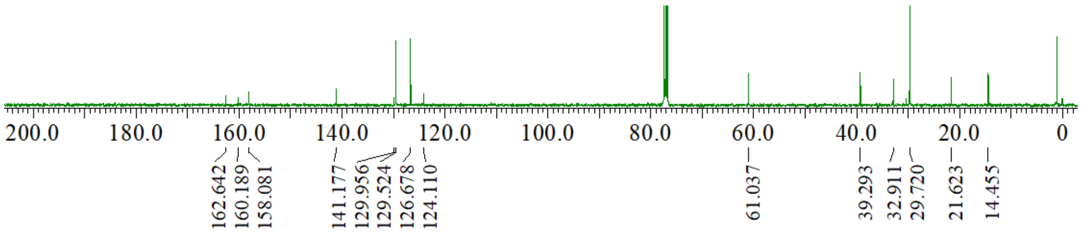

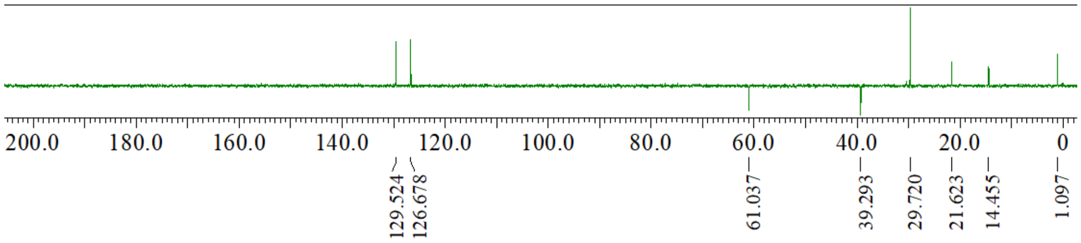

Ethyl 5-(trimethylsilyl)methyl-2-(4-methylphenyl)oxazole-4-carboxylate (**5d**)

^{1}H NMR (CDCl$_3$)

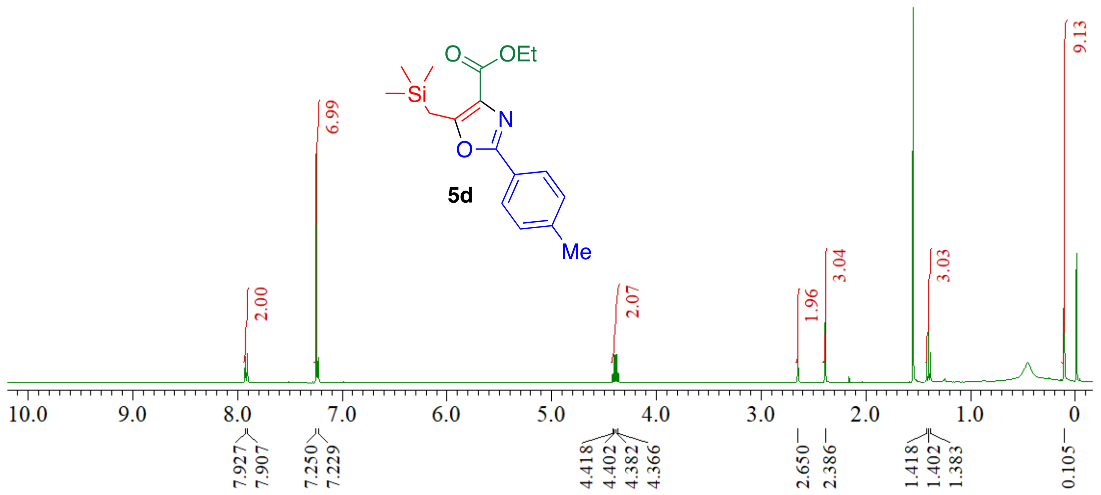

^{13}C{^{1}H} NMR (CDCl$_3$)

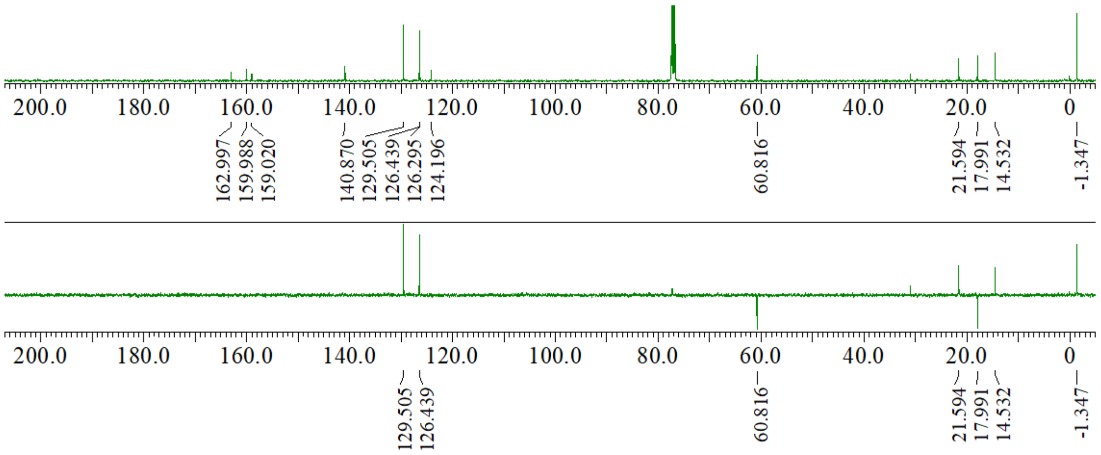

Ethyl 2-ethyl-5-(phenylmethyl)oxazole-4-carboxylate (**5e**)

^{1}H NMR (CDCl$_3$)

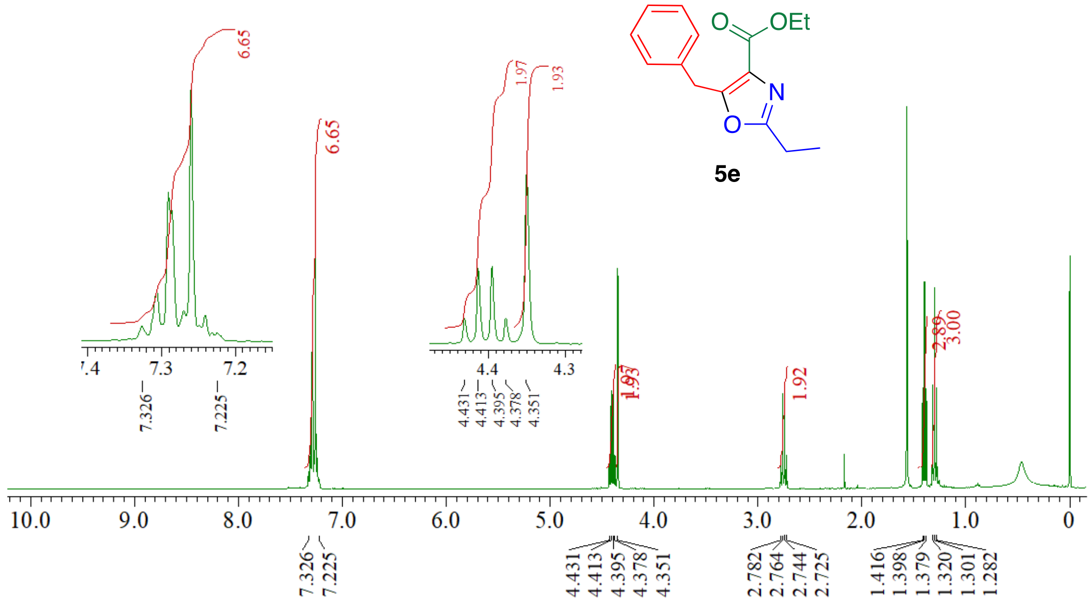

^{13}C{^{1}H} NMR (CDCl$_3$)

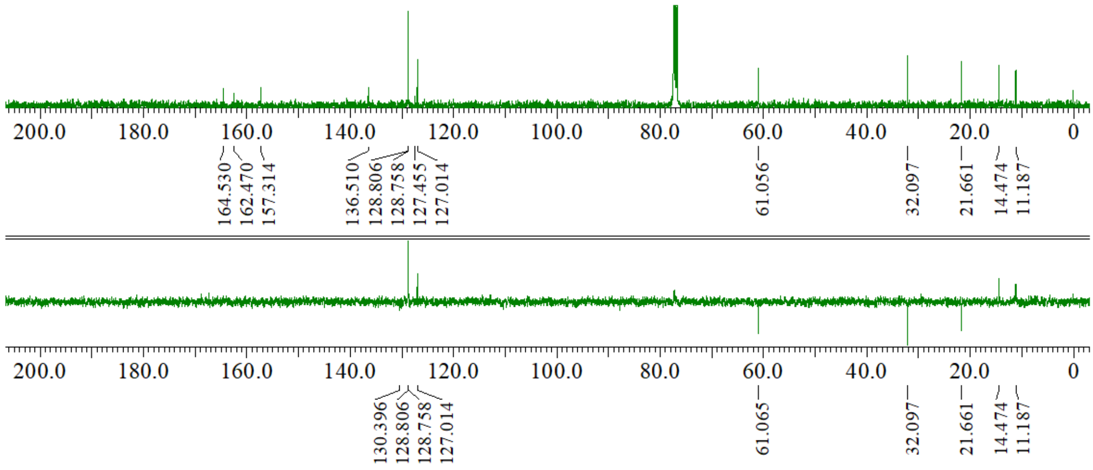

Diethyl 2-benzoylmethyl-2-[(4-methylbennzoyl)amino]propanedioate (**9**)
^{1}H NMR ($CDCl_3$)

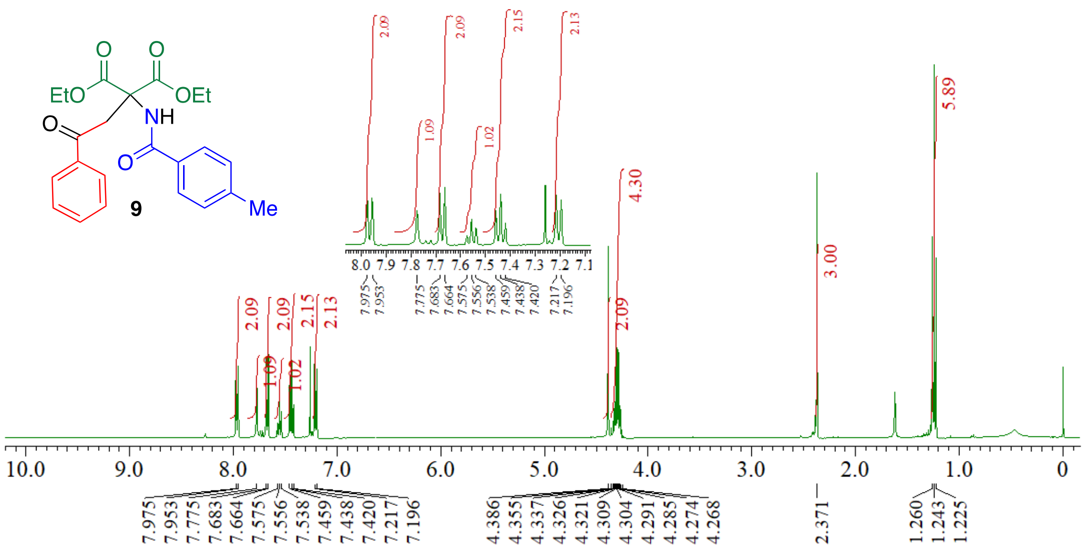

^{13}C{^{1}H} NMR ($CDCl_3$)

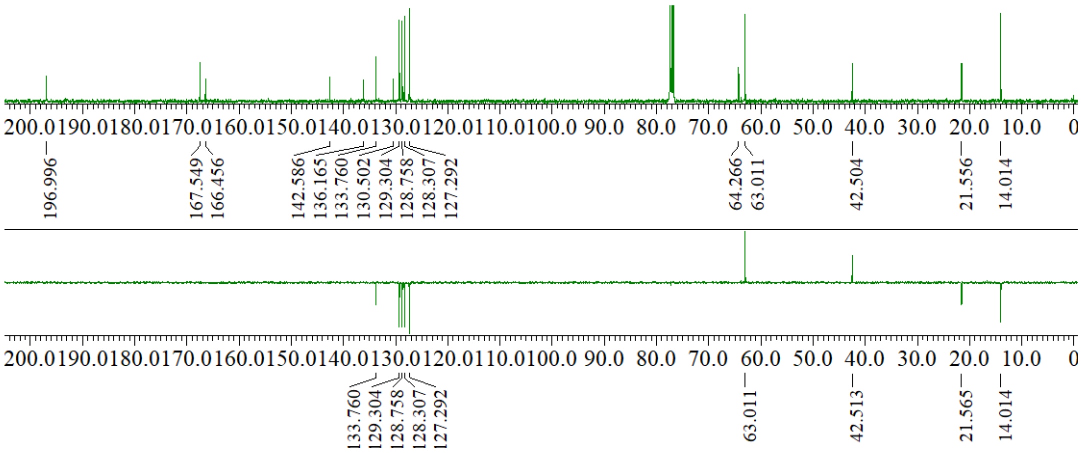

Supplement: File 1 — Spectral data for 4, 5, and 9 as well as 1H and 13C NMR spectra. [file Beilstein_J_Org_Chem-20-2827-s001.pdf]
